# The effect of PEGylated iron oxide nanoparticles on sheep ovarian tissue: An ex-vivo nanosafety study

**DOI:** 10.1016/j.heliyon.2020.e04862

**Published:** 2020-09-06

**Authors:** Sareh Karimi, Seyed Nasrollah Tabatabaei, Arno C. Gutleb, Marefat Ghaffari Novin, Alireza Ebrahimzadeh-Bideskan, Zahra Shams Mofarahe

**Affiliations:** a Department of Biology and Anatomical Sciences, School of Medicine, Shahid Beheshti University of Medical Sciences, Tehran, Iran; b Department of Medical Nanotechnology, School of Advanced Technologies in Medicine, Tehran University of Medical Sciences, Tehran, Iran; c Department of Pediatrics, Physiology and Pharmacology, University of Montreal, Montreal, QC, Canada; d Department of Environmental Research and Innovation (ERIN), Luxembourg Institute of Science and Technology (LIST), Esch s/Alzette, Luxembourg; e Department of Anatomy and Cell Biology, School of Medicine, Mashhad University of Medical Sciences, Iran

**Keywords:** Iron oxide nanoparticle, PEG, Silica, Ovary, Oxidative stress, Coatings, Materials safety, Antioxidant, Lipid peroxidation, Tissue culture, Nanotechnology, Toxicology, Reproductive system

## Abstract

Today, nanotechnology plays an important role in our ever-continuous quest to improve the quality of human life. Because of their infinitesimal size, nanostructures can actively interact and alter cellular functions. Therefore, while the clinical benefits of nanotechnology may outweigh most of the associated risks, assessment of the cytotoxicity of nanostructures in respect to cells and tissues early in product development processes is of great significance. To the best of our knowledge, no such assessment has been performed for nanomaterials on the ovarian cortex before. Herein, silica-coated, PEGylated silica-coated, and uncoated iron oxide nanoparticles (IONP) with core diameter of 11 nm (±4.2 nm) were synthesized. The oxidative stress in cultured ovarian tissue exposed to the various IONP was subsequently assessed. The results indicate that among the four groups, uncoated IONP induce the most oxidative stress on the ovarian cortex while tissues treated with PEGylated IONP exhibit no significant change in oxidative stress.

## 1 Introduction

Today, medical nanotechnology is already applied to a wide spectrum of treatments for countless patients around the world. Preventive strategies using nano-formulated vaccines, multimodal contrast agents for early detection of malignancies in medical imaging, intelligent biosensors for disease diagnosis [1], smart drug and gene delivery systems [2], and numerous therapeutic applications have allowed nanotechnology to secure itself a respectable place in modern medicine. However, as with most arising technologies, rapid advancements in this field raise serious biosafety concerns [3, 4]. Among the many types of nanomaterials, magnetic nanoparticles, and among those, iron oxide nanoparticles (IONP) have been particularly attractive for medical applications. These particles are extremely versatile, relatively biocompatible [5], the IONP have multifunctional applications in the fields of MRI, target-specific drug delivery, gene therapy, cancer treatments, in vitro diagnostics and in ex-vivo techniques such as cryopreservation [6]. Nevertheless, these nanostructures may have harmful effects due to the presence of iron as a redox agent, which releases reactive oxygen species and causes oxidative stress [7], DNA [8] and protein damage [9], as well as changes in the mitochondrial membrane potential [10].

The ovary is the most important and arguably the most delicate female reproductive organ and has two important anatomical and physiological parts: the medulla contains arteries, veins, and lymphatic veins; and the cortex protects oocyte-containing follicles as female fertility reserves. Among the important functions of these follicles is the production of hormones that ensure a women's health [11]. Unlike the testis, ovaries do not have a blood-tissue-barrier [12]. In addition, the main artery that feeds into the ovary branches from the aorta [13].

Silicon dioxide or silica is among popular coating agents for a variety of medical applications [14] that can increase colloidal stability and decrease the interaction between nanoparticles [15]. Silica coating is also employed as an alternative antifouling approach to polyethylene glycol or PEG [16]. PEG is a very popular polymer that is hydrophilic, non-immunogenic, and non-antigenic. It has a neutral charge and due to its rigidity for binding to plasma proteins, it is used as an effective coating agent to increase biological half-life for drug delivery purposes [17]. Due to the increasing use of IONP in medical applications, there is a serious concern about adverse effects of these nanoparticles on different tissues and cells [18, 19]. Yet, reports on biosafety of nanomaterials in the female reproductive organ are essentially non-existent [20].

In the present study, our aim has been to determine the toxicity effects of IONP under different coatings in the ovarian tissue. These nanoparticles are considered biocompatible and biodegradable and are being widely used in pre-clinical and clinical research. This study serves to raise awareness about the importance of such information and suggests that the risks associated with the use of nanomaterials may be contained by proper surface coating.

## 2 Material and method

### 2.1 Experimental design

The present study was designed to evaluate the effect of IONP on cultured sheep ovarian tissue. Particle penetration, oxidative stress and follicle viability were assayed. The ovarian samples were divided into four groups: i) untreated (n = 3), ii) cultured with uncoated Fe_3_O_4_ (n = 3), iii) cultured with Fe_3_O_4_@Silica (n = 3), and iv) cultured with Fe_3_O_4_@Silica@PEG (n = 3). Based on our preliminary findings, the chosen concentration (10 mg Fe/ml) for this study was enough to allow thorough assessment of the IONP effects in ex-vivo tissue in culture [6, 21]. Samples were randomly cultured for 24 h. The morphology of tissues was evaluated by Hematoxylin and Eosin (H&E) staining, follicle viability was assayed by neutral red staining, penetration profile of IONP was determined by Prussian blue staining, and to evaluate the oxidative stress, biochemical assessments were performed. To ensure the quality of tissue in culture, all the assessments were performed on fresh tissue samples, the results showed no significant difference with the untreated group (the results are not shown). This study was approved by the Shahid Beheshti University of Medical Sciences Ethics Committee (IR.SBMU.MSP.REC.13970435).

### 2.2 IONP synthesis and characterization

Synthesis of IONP was done according to a method explained by Chen et al in 2010 [22]. Briefly, we added 2 g of FeCl_3_ and equal amount of FeSO_4_ in 1-liter of ultra-distilled water at room temperature. Then, we used a magnetic stirrer to completely mix the solution. The solution was then deoxygenated by ultra-pure nitrogen gas bubbling for at least 15 min. To reach a pH of 9, we added ammonium hydroxide. The precipitated IONP were isolated by a magnet and washed three times with ethanol and were kept under oxygen free conditions.

In order to coat with silica, particles were dispersed in deionized water, then pH of the solution was adjusted to 9–10. Then, 1 ml tetraethoxysilane was added to the IONP solution and stirred for 3 h. To PEGylate the particles, 15 ml ethanolic Fe_3_O_4_@Silica suspension, containing 125 mg dry IONP and 729 μL of PEG-silane were added and stirred for 7 h. Then, the Fe_3_O_4_@Silica@PEG were separated by centrifugation at 15,000 rpm for 30 min [23].

**Figure 1:**
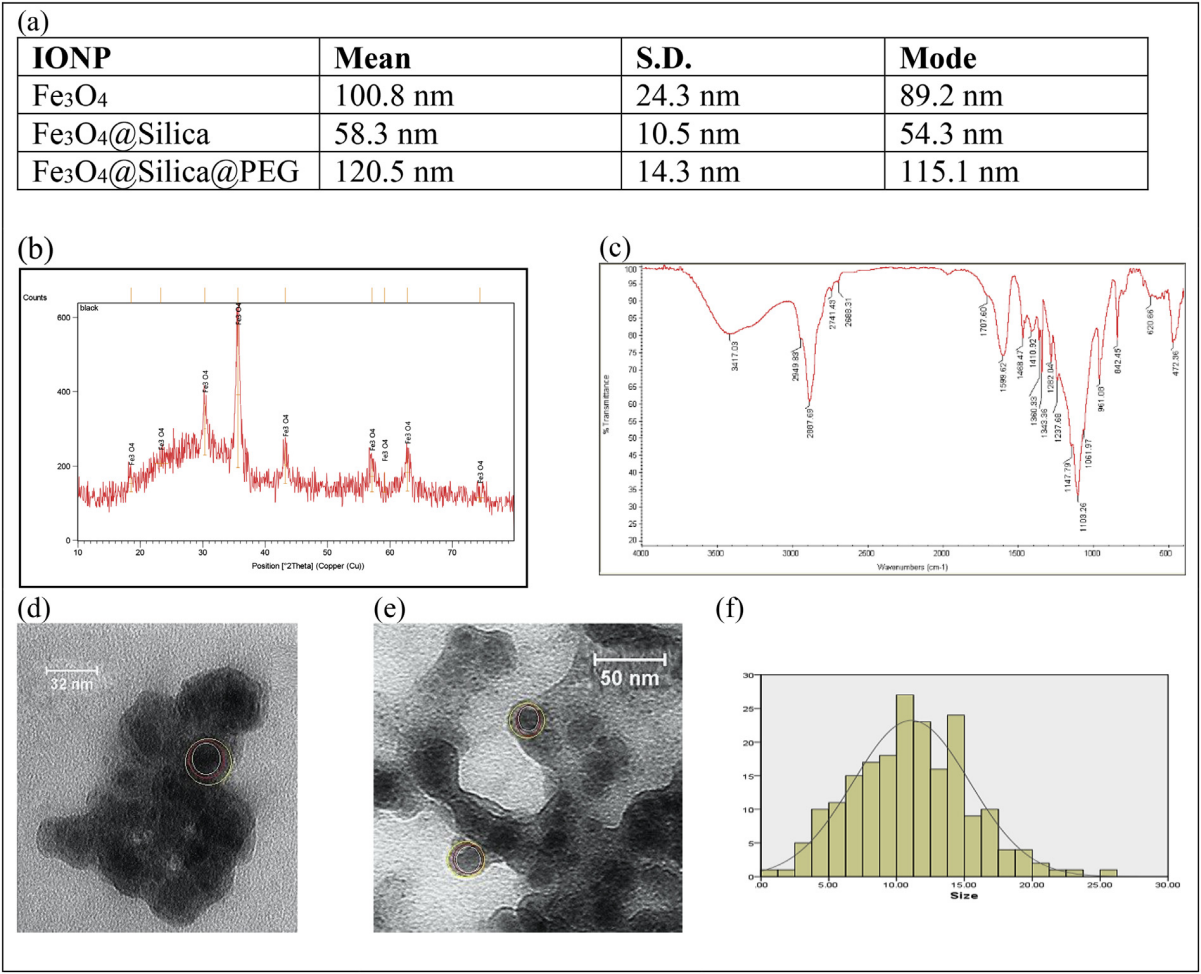
(a) DLS - The hydrodynamic diameter of IONP. (b) XRD of IONP. (c) FTIR of Fe3O4@Silica@PEG. (d and e) TEM of Fe3O4@Silica@PEG, the white circle is the Fe3O4, the red circle is the silica coat and the yellow circle is the PEG. (f) Core particle size distribution were 11 nm (±4.2 nm) in diameter.

After synthesis and coating, the IONP were characterized with Dynamic Light Scattering (DLS) (Figure 1(a)) and Zeta-potential (SZ-100 Nanoparticle Analyzer – Horiba, Japan), X-Ray Diffraction (XRD) (Philips PW 1730, Germany) (Figure 1(b)), Fourier Transform Infrared Spectroscopy (FTIR) (Thermo Nicolet AVATAR 370 FTIR) (Figure 1(c)), Transmission Electron Microscope (TEM) (Leo 912 Ab Omega, Zeiss) operated at 120 kV (Figure 1(d and e)).

### 2.3 Tissue collection & culture

Left ovaries from twelve sheep were procured from a slaughterhouse in Mashhad (Iran). We used bona fide excess tissue. No animals were specifically euthanized for our research. Ovaries were transported in HamsF10 supplemented with 50 mg/ml streptomycin and 60 IU/ml penicillin (Gibco) and 10% human serum albumin (HSA), at 4 °C. In the laboratory, the ovaries were washed three times with fresh sterile PBS and placed in sterile petri dishes containing HamsF10 supplemented with HEPES. Next, the medulla was removed and subsequently each cortex was cut into 24 to 25 slices with approximate dimensions of 1 mm (length)×1 mm (width)×1 mm (thickness). Sections were placed in fresh HamsF10 medium supplemented with 50 mg/ml streptomycin and 60 IU/ml penicillin and 10% HAS. The ovarian pieces were washed three times with HamsF10. Each slice was cultured individually in a 96-well culture plate for 24 h in 300 μl DMEM supplemented by 10% FBS (Fetal Bovine Serum, Gibco), 50 mg/ml streptomycin, and 60 IU/ml penicillin in incubator at 37 °C and 5% CO_2_ [24,25].

### 2.4 Histology assessment

Ten samples from each experimental group were fixed at room temperature by 10% formaldehyde. The samples were dehydrated, cleared and embedded in paraffin and sectioned with a microtome (Leitz 1512, Germany) at 5 μm thickness. Every 10th section was mounted on a glass slide and stained [26].

#### 2.4.1 Hematoxylin & Eosin staining

The glass slides were stained with H&E and mounted. Each section was then observed through a light microscope (Zeiss, Germany) and the histology of tissue in preparation time was assessed (X40 objective).

#### 2.4.2 Prussian blue staining

To prepare the Prussian blue staining material, a solution of 10% Potassium Ferrocyanide and 20% hydrochloric acid were equally measured and mixed in distilled water. Immediately after, the tissue on the glass slide was stained with the resulting solution for 20 min at room temperature and counterstained with nuclear fast red for 5 min. In the obtained microscopic images, the detected blue spots are iron, and pink spots are the cellular nuclei (X100 objective) [27].

#### 2.4.3 Neutral red staining

To assess the viability of the ovarian follicles, neutral red staining was used. Generally, living cells can incorporate and bind with neutral red in cellular lysosomes [28]. For this technique, five ovarian fragments from each group were immersed in culture medium with 50 μg/ml of neutral red solution (2-amino-3methyl-7-dimethyl-aminophenazoniumchloride, Sigma-Aldrich, USA) and incubated for 4 h at 37 °C and 5% CO_2_. After washing with PBS, the fragments were placed on glass slides and a cover slip was pressed on the softened tissue samples. Living (stained red) as well as dead (not stained) follicles were counted by a light microscope (×20 objective) [29].

### 2.5 Biochemical assays

MDA level is an index to evaluate lipid peroxidation injury. MDA reacts with thiobarbituric acid (TBA) as a TBA reactive substance (TBARS) and produces a red complex. Briefly, the samples were homogenized and 2 mL of a solution containing TBA, trichloroacetic acid, and hydrochloric acid was added. The solution was then boiled in a water bath for 40 min. After cooling, the solution was centrifuged at 1000 g for 10 min. The absorbance was read at 535 nm by Elisa reader (Epoch, BioTek, US). The MDA concentration (*C*) was calculated according to following equation [30].

C = Absorbance/1.56 × 10^5^

SOD activity was measured by a procedure described by Madesh and Balasubramanian [30]. A colorimetric assay involving generation of superoxide by pyrogallol auto-oxidation and the inhibition of superoxide-dependent reduction of the tetrazolium dye, 3-(4,5-dimethylthiazol-2-yl) 2,5-diphenyltetrazolium bromide (MTT) to its formazan by SOD was measured at 570 nm. One unit of SOD activity is defined as the amount of enzyme causing 50% inhibition in the MTT reduction rate.

CAT activity was measured according to the Aebi method with some modifications [31]. The aim of this assay is to determine the rate of hydrogen peroxide decomposition. Reduction in absorbance (at 240 nm) per minute was determined by UV-Vis spectrophotometer (Cecil, UK) and the rate of the enzyme was calculated.

### 2.6 Statistics analysis

Data were quantified using ImageJ software and were analyzed using a one-way ANOVA with Bonferroni post hoc analysis with GraphPad Prism software (version 8.0; GraphPad Software, San Diego, CA). A Student's t-test (unpaired) was used for studies comparing two groups. Statistical significance was established a priori at *p* < 0.05.

## 3 Result

### 3.1 Physico-chemical characterization of IONP

The particles were extensively characterized using XRD (Figure 1(b)), based on DLS the mean hydrodynamic diameter of Fe_3_O_4_ without coating, Fe_3_O_4_@Silica and Fe_3_O_4_@Silica@PEG respectively, was 101.1 nm, 58.3 nm and 120 nm (Figure 1(a)), the larger size of Fe_3_O_4_ was due to agglomerated Fe_3_O_4_ particles during the measurement time, FTIR confirmed the PEGylation of IONP (Figure 1(c)), TEM (Figure 1(d and e)) confirmed the coating IONP and using TEM images, the size of hundreds of particles was measured by ImageJ software, and the histogram curve was plotted by IBM SPSS statistic 22 software. Average size of the IONP is 11 nm (±4.2 nm) (Figure 1(f)).

### 3.2 IONP penetration

**Figure 2:**
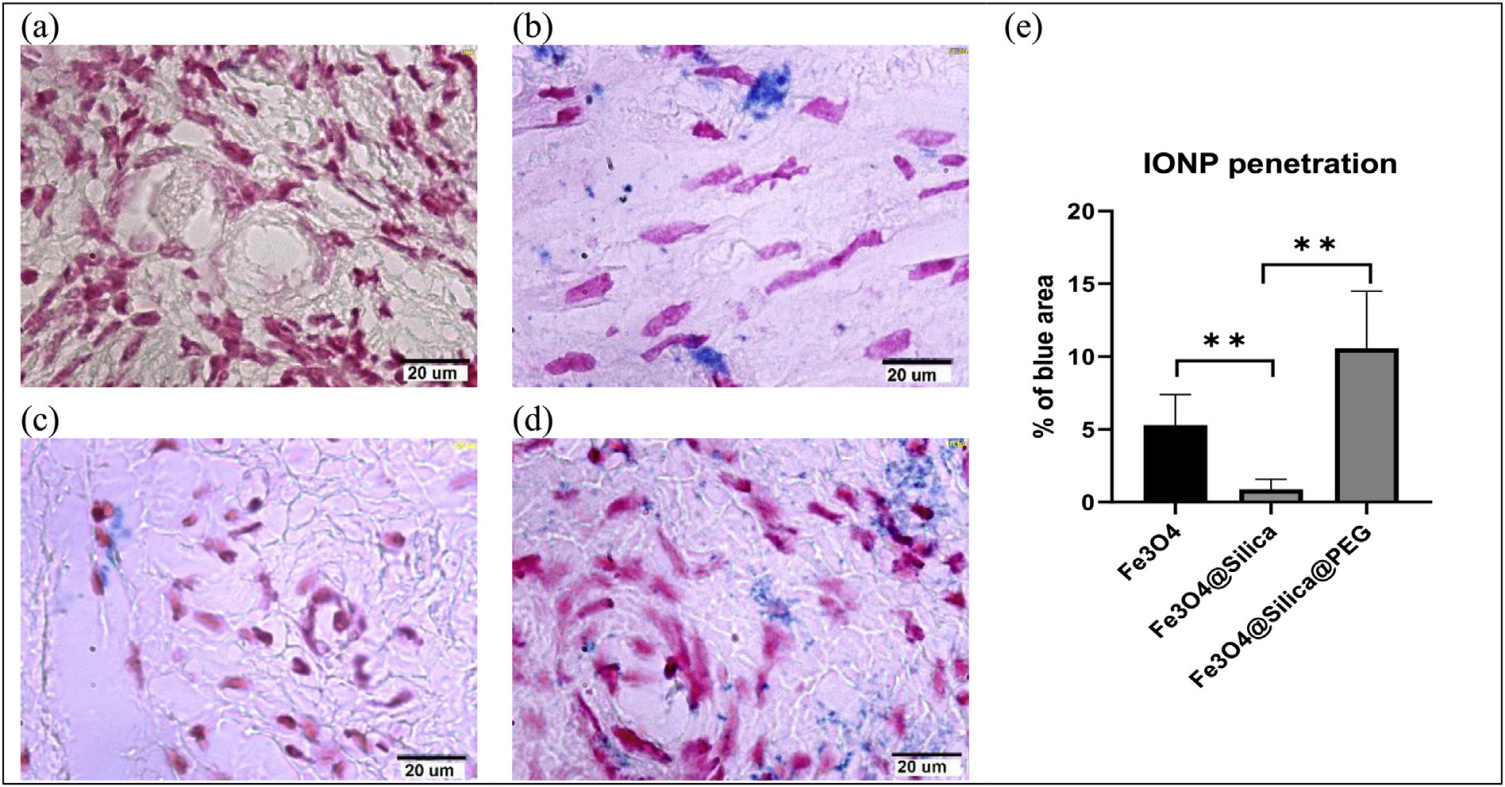
IONP penetration over 24 h. Prussian blue staining in (a) untreated, (b) cultured with uncoated Fe_3_O_4_, (c) cultured with Fe_3_O_4_@silica, and (d) cultured with Fe_3_O_4_@Silica@PEG groups. The pink spots indicate cell nuclei and the blue spots are IONP, (Magnification of 100X). (e) A quantitative evaluation using ImageJ software shows the percentage of blue spots in each image (∗∗ indicates *p* < 0.01).

Prussian blue staining results (Figure 2(a-d)) show that uncoated Fe_3_O_4_ and Fe_3_O_4_@Silica@PEG particles exhibit higher levels of tissue penetration than Fe_3_O_4_@Silica particles (Figure 2(e); *p* < 0.01).

### 3.3 Biochemical assays

**Figure 3:**
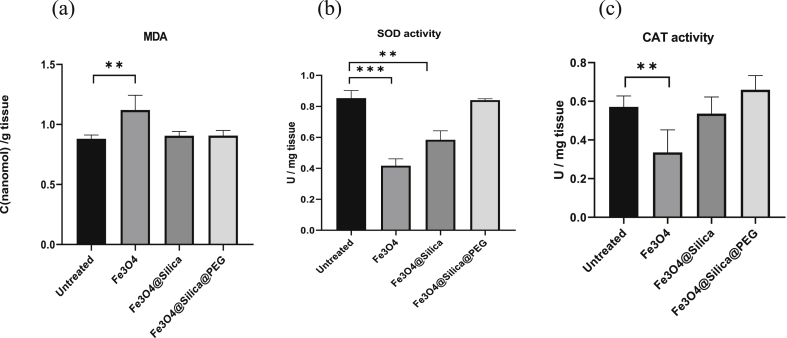
Oxidative stress of cultured tissue. (a) MDA levels (C: concentration), (b) SOD activity (U: Unit), and (c) CAT activity (U:Unit)of tissue cultured with various IONP coatings compared to untreated tissue (∗∗ indicates *p* < 0.01, ∗∗∗ indicates *p* < 0.001).

Results show that, compared to the untreated tissue, cultured ovarian tissue with Fe_3_O_4_ displayed elevated levels of MDA (Figure 3(a); *p <* 0.01), decreased levels of SOD activity (Figure 3(b)*; p* < 0.001), and decreased levels of CAT activity (Figure 3(c); *p* < 0.01). The MDA and CAT activity levels of samples treated with Fe_3_O_4_@Silica did not significantly differ from untreated tissue (Figure 3(a and c)). Nevertheless, treated samples expressed decreased levels of SOD activity (Figure 3(b); *p* < 0.01) compared to the untreated tissue. Samples treated with Fe_3_O_4_@Silica@PEG did not inflict any significant oxidative stress on the tissue (Figure 3(a-c)).

### 3.4 Follicular viability assessment

**Figure 4:**
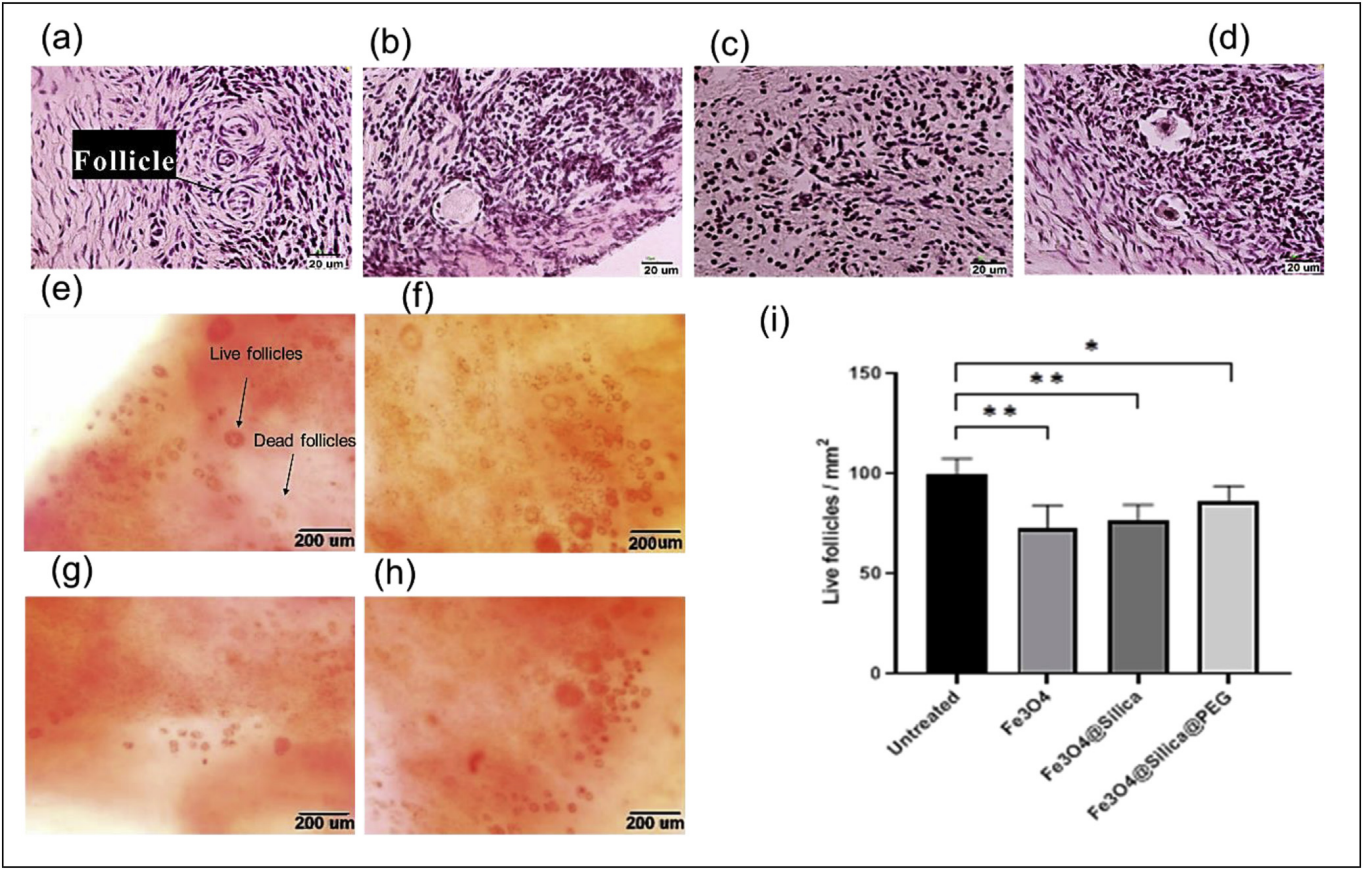
Live follicle density. H&E staining to show the follicles in ovarian tissue in (a) untreated, (b) cultured with uncoated Fe_3_O_4_, (c) cultured with Fe_3_O_4_@silica, and (d) cultured with Fe_3_O_4_@Silica@PEG groups (magnification of 40X). Neutral red staining in in (e) untreated, (f) cultured with uncoated Fe_3_O_4_, (g) cultured with Fe_3_O_4_@silica, and (h) cultured with Fe_3_O_4_@Silica@PEG groups. The stained follicles (red) are alive and non-stained follicles are dead (magnification of 20X). (i) Live follicles per mm^2^ of ovarian tissue as evaluated by neutral red staining (∗ indicates *p* < 0.05, ∗∗ indicates *p* < 0.01).

The accuracy of tissue preparation was evaluated by H&E staining, the thickness of tissue and primordial follicle density (live and dead) in all groups was similar and no damage was done to the tissue during the preparation process (Figure 4(a-d)). To investigate the number of live follicles however, neutral red staining was employed (Figure 4(e-h)). Neutral red staining results depicted in Figure 4 indicate that tissue treated with uncoated and Fe_3_O_4_@Silica exhibit lower follicle viability compared to Fe_3_O_4_@Silica@PEG particles.

## 4 Discussion

As we know, IONP have many applications in medicine including: enhance targeting for drug delivery [32], hyperthermia to complement treatment of various tumor types [33], promote transient opening of the blood-brain barrier [34], enhance MRI contrast for medical imaging [35]. In addition, IONP-based drugs are FDA-approved to treat iron deficiency [36, 37]. The ovary is the most important and arguably the most delicate female reproductive organ that feeds from the main artery which in turn stems from ovarian branches of the aorta [13]. Therefore, according to the anatomical condition of the ovary, following systemic administration, nanomaterials can easily reach the ovarian cortex and accumulate. Nevertheless, there are no studies that look into the in-vivo, in-vitro and ex-vivo effects of IONP on the ovary.

In this study, the toxicity of coated (silica and PEG) and uncoated IONP were examined. Other than reducing dipole-dipole interactions between IONP [38], surface modification of particles limit toxicity [39], increase vascular circulation half-life [14], and enhance targeting capabilities [40]. PEG is a very popular polymer that is hydrophilic, non-immunogenic, and non-antigenic. It has a neutral charge and due to its rigidity for binding to plasma proteins, it is used as an effective coating agent to increase biological half-life for drug delivery purposes [17]. Silica is also among conventional choices of particle coatings that due to its enhanced cellular penetration and uptake is often used [41].

Based on the DLS results (Figure 1(a)), the hydrodynamic diameter of uncoated Fe_3_O_4_ was larger than Fe_3_O_4_@Silica, suspected due to agglomeration. Fe_3_O_4_@Silica@PEG had the largest reported hydrodynamic diameter among all samples. This is mainly due to the fact that PEG is a hydrophilic molecule and interacts with water molecules in an aqueous solution via hydrogen bonds. This interaction leads to a wider scattering of light and subsequently a larger particle size reading in the DLS apparatus [42].

Prussian blue staining results show that uncoated Fe_3_O_4_ and Fe_3_O_4_@Silica@PEG particles exhibit higher levels of tissue penetration than Fe_3_O_4_@Silica particles (Figure 2(e)). Rascol et al. investigated the real time PEG and silica coated IONP cell penetration and demonstrated silica enhance the passage through the cell membrane and silica coated IONP observed in intracellular vesicles [43], this data confirm by another studies [41], and since PEG cause hydrophilicity so it cannot transfer to cell easily [42] and stop in extracellular space.

Based on our TEM results, uncoated IONP are smaller in size (Figure 1(d -f)) and can penetrate the tissue [44, 45]. Once particle penetration was confirmed, the physiology of the cultured tissue exposed to IONP was examined. Specifically, the distribution of ROS, a natural byproduct of cellular metabolism in mitochondria was assessed under external stress. Generally, elevation of ROS increases MDA levels which is an indication of oxidative stress [46]. On the other hand, SOD and CAT are ROS scavengers which serve to suppress the adverse effects of ROS such as cellular necrosis [47]. Based on our data (Figure 3(a-c)), uncoated IONP elevated levels of MDA while decreasing levels of SOD and CAT activity. These results indicated that uncoated IONP promote oxidative stress in the ovarian cortex. This could be due to the release of iron from uncoated particles onto the tissue which then induces Fenton-like reactions, free radicals, and oxidative injury [39]. The MDA and CAT activity in the samples treated with Fe_3_O_4_@Silica did not significantly differ from that of untreated tissue. Nevertheless, treated samples expressed decreased levels of SOD activity (Figure 3(a-c)) compared to the untreated tissue. This provides substantial evidence that silica assists in ROS formation and consequently can lead to reduced cellular viability [48]. Most importantly, we show that samples treated with Fe_3_O_4_@Silica@PEG did not inflict any significant oxidative stress on the cells (Figure 3(a-c)). The physicochemical properties of IONP play a crucial role in determining their cytotoxicity. In our study, Fe3O4@Silica and Fe3O4@Silica@PEG were in the same range in terms of size [49]. Their different behaviors in tissue may therefore be due to other factors such as increased penetration of silica [43] or higher molecular weight and grafting density of PEG that reduces interactions between proteins and nanoparticle surfaces [50]. Moreover, hydrophobic polymers interact better with proteins [51].

Oxidative stress and ROS are influential factors on the quality of an oocyte; indeed, ROS play an important role in cell signaling for initial and continued meiosis in the oocyte [52]. However, increased ROS levels disturb the maturation of oocytes and affect the growth and development of follicles, in a way that oxidative stress can reduce the number of available follicles [53], possibly resulting in female infertility. According to our results (Figure 4(e-h)), the number of live follicles per mm^2^ of the ovarian tissue decreased in all experimental groups compared to the untreated tissue, as assayed by the neutral red staining test. The viability of follicles was lower in the Fe_3_O_4_@Silica and Fe_3_O_4_ treated groups (Figure 4(i)). Fe_3_O_4_@Silica@PEG particles that had displayed higher levels of particle penetration (Figure 2(e)), resulted in better survival of follicles/mm^2^ in culture (Figure 4(i)). Therefore, it seems that oxidative stress was less damaging to the Fe_3_O_4_@Silica@PEG treated tissue compared to the other experimental groups.

While the mentioned applications, and many more in development, are gaining legitimate grounds in the medical field, the harmful effects of IONP continue to be a concern. In particular, cytotoxicity assessments on a variety of cell lines [54] and tissue samples [55] suggest that IONP induce an alarming level of oxidative stress. These studies also reveal that the surface coating of IONP often dictate the level at which oxidative stress is applied [39]. A survey of relevant literature however indicates that, although routine in many organs, these assessments in the ovarian tissue are lacking.

## 5 Conclusion

In this study, we demonstrated that uncoated IONP and silica-coated IONP in comparison to PEGylated, silica-coated IONP induce more oxidative stress and therefore damage tissue by decreasing follicle numbers.

## Declarations

### Author contribution statement

S. Karimi: Conceived and designed the experiments.

S.N. Tabatabaei: Performed the experiments.

A.C. Gutleb: Analyzed and interpreted the data; Wrote the paper.

M.G. Novin: Conceived and designed the experiment; Wrote the paper.

A. Ebrahimzadeh-Bideskan and Z.S. Mofarahe: Analyzed and interpreted the data; Contributed reagents, materials, analysis tools or data.

### Funding statement

This work was supported by 10.13039/501100005851Shahid Beheshti University of Medical Sciences (Project No:15263).

### Competing interest statement

The authors declare no conflict of interest.

### Additional information

No additional information is available for this paper.
